# Community Characteristics Associated With Health System Penetration

**DOI:** 10.1177/10775587261469029

**Published:** 2026-09-28

**Authors:** Haoyu Bi, Mark Louie Ramos, Jeannette A. Rogowski, Charleen Hsuan

**Affiliations:** 1 The Pennsylvania State University, University Park, USA

**Keywords:** health disparities, underserved area, health system

## Abstract

Health systems have become a dominant form of health care organization in the United States, yet little is known about how their presence varies across communities. This study examined the association between the community-level characteristics and health system penetration, defined as the percentage of general acute care hospital beds affiliated with a health system in a geographic market. We found that communities with higher health system penetration tended to have lower levels of competition. In rural areas, greater insurance coverage was associated with greater penetration. In urban areas, communities without health system presence were more likely to have larger Black populations relative to White populations. However, among communities with some system presence, those with larger Black populations tended to have higher levels of penetration. Policymakers should adopt context-specific policies that encourage investment in underserved communities, promote equitable access to high-quality care, and guard against the potential adverse effects of consolidation.

## Introduction

Over the past 2 decades, health care providers in the United States have undergone substantial consolidation through several forms of integration, including horizontal consolidation (e.g., mergers between similar entities such as hospitals), vertical integration (e.g., hospitals acquiring physician practices), and cross-market consolidation (e.g., mergers between entities in different geographic areas). As these arrangements have expanded, integrated health systems have become a dominant form of health care organization. Hospitals and physician groups are increasingly vertically integrated within health systems (Godwin et al., 2026; Machta et al., 2020), although governance structures vary considerably both within and across systems (Ridgely et al., 2019). In 2016, 69.7% of U.S. nonfederal general acute care hospitals were affiliated with health systems, and these hospitals accounted for 88.2% of all hospital beds (Furukawa, Machta, et al., 2020). System-affiliated hospitals and physicians accounted for a large proportion of Medicare admissions (Beaulieu et al., 2023).

The impact of the growing dominance of health systems on patients has been well studied and appears mixed. On one hand, health system may offer potential benefits, including greater care integration and active engagement in care redesign activities, which may in turn support better quality of care (Colla et al., 2020; Harvey et al., 2020; Ridgely et al., 2020; Scanlon et al., 2020; Singer et al., 2020). For example, one study found that clinics acquired by health systems had diabetes care performance that was 2.3 percentage points higher than it would have been had they remained independent (Crespin et al., 2016). On the other hand, another study found that most health systems did not consistently achieve either high or low performance on quality measures (Kranz et al., 2020). Similarly, a systematic review of vertical integration concluded that such integration may improve some quality-of-care measures but was also associated with no difference or lower efficiency (Machta et al., 2019). By contrast, prior studies have more consistently found that health system consolidation is associated with higher prices and costs (Carlin et al., 2017; Henke et al., 2018; Lewis & Pflum, 2017).

Despite the growing prevalence of consolidation, health system presence varies substantially across geographic areas. For example, the share of hospital beds affiliated with health systems ranges from 47.4% in Wyoming to 98.2% in Hawaii (Furukawa, Machta, et al., 2020). However, little is known about whether certain types of areas are disproportionately served by health systems. Economic theories of firm behavior identify expected profits from market entry as a central driver of market participation (Dixit, 1980; Geroski, 1995). From a profit-seeking perspective, health systems may be more likely to expand into communities that offer stronger financial returns, or in rural communities where hospital acquisitions permit systems to secure dominant market positions that can be leveraged in negotiations with insurers. Existing evidence offers some support for both strategies. Supporting the first strategy, hospitals generally have higher operating margins in metropolitan areas than in nonmetropolitan areas (KFF, 2024). Therefore, the majority of system acquisitions occurred in metropolitan areas relative to nonmetropolitan areas (Furukawa, Kimmey, et al., 2020), and the growth of system-affiliated hospitals increased faster in the former than in the latter (Oyeka et al., 2018). Supporting the second strategy, rural hospitals, particularly those that are larger, for-profit, less profitable hospitals, and burdened with higher debt burdens, have increasingly been targeted by acquirers because these hospitals offer the potential to secure dominant market positions (Williams et al., 2020). Guided by this framework, we focus on two broad domains of community-level determinants of health system penetration: sociodemographic characteristics and the health care delivery environment. We hypothesize that health systems are more likely to expand into communities with more favorable sociodemographic profiles or stronger demand for health care services.

Understanding whether certain communities are disproportionately served by health systems is especially important. Some communities may be particularly vulnerable to the adverse effects of consolidation. For example, acquisitions of safety-net hospitals by health systems have sometimes led to reductions or eliminations of essential services (Wood et al., 2024), which reduce access to care for low-income residents in the hospitals’ catchment areas because of their disproportionate use of the acquired hospitals. Hospital mergers have also been linked to declines in health insurance take-up, with disproportionately larger effects among non-White populations than among White populations (Town et al., 2007). At the same time, health systems may also have the capacity to reduce health disparities within the communities they serve (Jiang et al., 2021; Wesson et al., 2019). These patterns underscore the importance of evaluating health system market characteristics from an equity perspective.

In this study, we examine which community-level characteristics are associated with health system penetration. We pay particular attention to whether disadvantaged communities, such as those with higher proportions of racial and ethnic minority residents or low-income residents, are more likely to experience lower health system penetration.

## New Contribution

This study is, to our knowledge, the first to evaluate the association between community characteristics and health system penetration. By integrating multiple public data sources to characterize several dimensions of community context, our study contributes new evidence on how health system penetration is distributed across U.S. communities. These findings can inform federal and state oversight, such as antitrust and regulatory strategies that consider not only market competition but also equity implications (Leibenluft, 2022), and guide future research into how consolidation differentially affects access, quality, and outcomes across diverse communities in an increasingly consolidated health care delivery environment.

## Method

### Data and Sample

To examine the association between community-level characteristics and health system penetration, the unit of analysis in this study was the geographic health care market. However, there is no single standard definition of a geographic health care market. Prior studies have used a range of market definitions, including Metropolitan Statistical Areas (David et al., 2002; Fulton, 2017), hospital referral regions (Cutler & Scott Morton, 2013), and floating catchment area methods (Delamater, 2013; Wan et al., 2012). Each of these alternatives involves tradeoffs. Metropolitan Statistical Areas are commonly used, but they exclude rural and micropolitan areas. Hospital referral regions are useful for capturing referral patterns for tertiary medical care, but they may be too large to reflect local community-level market conditions. Floating catchment area methods are flexible and can better approximate spatial access to nearby providers, yet they require custom market definitions and are difficult to harmonize with area-level sociodemographic measures. After considering strengths and limitations of these alternatives, and consistent with recent empirical work (Carroll et al., 2023; Crook et al., 2024; Fulton et al., 2022), we define health system markets using 2020 Commuting Zones (CZs). CZs are groups of counties based on journey-to-work patterns identified by the U.S. Census Bureau and are intended to represent integrated local labor markets (U.S. Department of Agriculture, Economic Research Service, 2026). They were well-suited to our study because they may serve as a proxy for patients’ willingness to travel for hospital services, capture markets across both urban and rural settings, and are constructed from counties, which allows county-level characteristics to be aggregated consistently to the market level.

The 2020 CZ data include 598 CZs nationwide, some of which cross state boundaries. Because hospital mergers, acquisitions, and other ownership transfers are often subject to state-specific notification or approval requirements, state boundaries remain relevant for health system activity. Therefore, following prior work by Escarce et al. (2022), we divided CZs spanning multiple states into distinct CZ–State units. We refer to these units as zones throughout the study. We then excluded zones without any hospitals. The final analytic sample consisted of 694 zones over 6 years, yielding a longitudinal panel of 4,144 zone-year units (see Supplemental Appendix I in the supplemental materials for the detailed sample selection process).

County-level sociodemographic characteristics were obtained from the U.S. Census Bureau and aggregated to the zone level. Hospital characteristics were obtained from the American Hospital Association (AHA) Annual Survey Database. Information on hospital system affiliation was obtained from the Agency for Healthcare Research and Quality (AHRQ) Compendium of U.S. Health System. The AHRQ Compendium defines a health system as an organization that includes at least one hospital and at least one physician group providing comprehensive services, linked through common ownership or joint management (Agency for Healthcare Research and Quality, 2025). We relied on the AHRQ Compendium definition because, unlike the self-reported system affiliation measure in the AHA Annual Survey, it is based on a consensus definition developed by a broad panel of national experts and derived from multiple data sources.

### Variables

The outcome variable was health system penetration. We focused specifically on health system penetration through ownership or joint management of general acute care hospitals. Health system penetration was defined as the percentage of general acute care hospital beds in a zone that were affiliated with a health system in a given year. This bed-weighted measure reflects the extent to which inpatient hospital capacity within a community was controlled by health systems. We grouped the independent variables into two domains based on our conceptual focus: sociodemographic characteristics and the health care delivery environment, as described in the following text.

Sociodemographic factors capture the socioeconomic and demographic context of each zone. These measures included the percentage of population aged 65+, the percentage of adults aged ≥25 years with at least a high school education, the percentage of residents enrolled in Medicaid, the percentage of residents with private insurance, the percentage of residents living below the federal poverty level, and whether the zone was classified as a high racial and ethnic minority zone (“minority zone”). We included these measures because these characteristics may shape both the demand for health care services and the broader structural conditions that may be associated with health system penetration. For example, a higher proportion of residents with private insurance may offer a more stable source of revenue, educational attainment may be associated with health literacy and patterns of health care engagement, and racial and ethnic composition may capture broader structural and geographic inequalities relevant to local market conditions. We defined minority zones as those in which the proportion of White residents fell below the 25th percentile of the national distribution. These zones were then categorized as high-Black, high-Hispanic, or high-other based on the largest minority population group within the zone.

The health care delivery environment captured features of the local delivery system that may affect the attractiveness of a zone to health systems. These measures included the number of hospitals in the zone, U.S. regions (U.S. Census Bureau, 2021), hospital market concentration, and rurality. Hospital market concentration was measured using the Herfindahl–Hirschman Index (HHI), a widely used measure of market concentration. The HHI was calculated as the sum of squared market shares and ranges from 0 to 10,000, with higher values indicating greater market concentration and lower competition (U.S. Department of Justice, Antitrust Division, 2024). In calculating the HHI, hospitals owned by the same health system were treated as a single entity, and market shares were based on staffed hospital beds within the zone. Rurality was defined using the 2023 National Center for Health Statistics (NCHS, 2025) Urban–Rural Classification Scheme for Counties. This scheme was chosen because it is a standardized and widely used county-level classification in health services research. We first created a county-level dataset containing zone identifiers, total population, and NCHS classification codes. Counties with NCHS Codes 1 through 4 were classified as urban, and those with Codes 5 and 6 classified as rural. For each zone-year, we calculated a population-weighted rural share by dividing the total population living in rural counties by the total population of the zone. Zone-years were classified as rural if 50.0%–100% of the population lived in rural counties and as urban if less than 50.0% of the population lived in rural counties (Ratcliffe et al., 2016).

### Statistical Analysis

We first compared characteristics across different levels of system penetration in solo-hospital zones and multi-hospital zones separately. Differences across groups were assessed using chi-square tests for categorical variables and Kruskal–Wallis tests for continuous variables, as appropriate.

For multivariable analysis, we stratified zones into solo-hospital zones and multi-hospital zones because the structure of the outcome differed substantially across these two settings. In solo-hospital zones, health system penetration was binary because the only hospital in the zone was either system-affiliated or not. For these zones, we estimated a logistic regression model with year fixed effects and robust standard errors clustered at the zone level.

In multi-hospital zones, health system penetration ranged from 0 to 1 and included a substantial number of observations at the boundary values of zero and one. To account for this distribution, we estimated zero-one inflated beta (ZOIB) regression models. The ZOIB model consisted of three components: a logit model for the probability that penetration equaled zero, a beta regression for observations strictly between zero and one, and a logit model for the probability that penetration equaled one. This approach preserves the full structure of the data by modeling boundary values and fractional outcomes within a single coherent framework (Ocaña-Riola et al., 2021). Covariates in these models included both sociodemographic characteristics and the health care delivery environment measures. Considering the motivations for health system expansion may differ between urban and rural areas (Oyeka et al., 2018; Williams et al., 2020), we further stratified regression models by rural and urban zones. In general, if the mechanism underlying system expansion were consistent in all markets, we would expect coefficient signs to move in opposite directions in the zero-inflation and one-inflation components, whereas the sign in the beta component would more closely resemble that in the one-inflation component. Divergences from this pattern may indicate that system expansion varies based on whether there is already a system present in the market.

In sensitivity analyses, we used a state-relative definition of minority zones, given substantial cross-state variation in demographic composition (U.S. Census Bureau, 2023) and the relevance of state-specific regulatory and policy contexts for hospital ownership changes. Under this alternative definition, zones with a proportion of White residents below the 25th percentile of the state-specific distribution were classified as minority zones and further categorized as high-Black, high-Hispanic, or high-other according to the largest minority population group within the zone.

All regression models included year fixed effects to account for common temporal shocks and secular trends across zones. Standard errors were clustered at the zone level to account for within-zone correlation over time. An α level of 0.05 was used for all statistical two-tailed tests. Analyses were conducted using Stata 18.0. Institutional Review Board approval was not required for this study as it involved analysis of de-identified secondary data and did not constitute human subject research.

## Results

Figure 1 illustrates the geographic distribution of health system penetration across zones in 2016 and 2023. The maps suggest an overall increase in health system penetration over time, with a growing number of zones moving toward full penetration by 2023. This pattern appears especially pronounced in the South and Northeast, where zones with high levels of system penetration became more common over the study period. Table 1 summarizes descriptive characteristics in solo-hospital zones and multi-hospital zones. Among solo-hospital zones, 358 zone-year observations (52.3%) had a system-affiliated hospital. Urban zones accounted for 19.6% of zones with a system-affiliated hospital, compared with 5.8% of zones with a non-system-affiliated hospital. Among multi-hospital zones, 290 zone-year observations (8.4%) had no health system penetration, and 985 (28.5%) had full penetration. All community characteristics differed significantly across the three levels of system penetration. Educational attainment and private insurance coverage were higher in zones with full penetration. Of the penetrated zones, 10.5% of partially penetrated zones and 9.2% of fully penetrated zones were high-Black, compared with only 4.8% of zones with no penetration.

**Figure 1.**
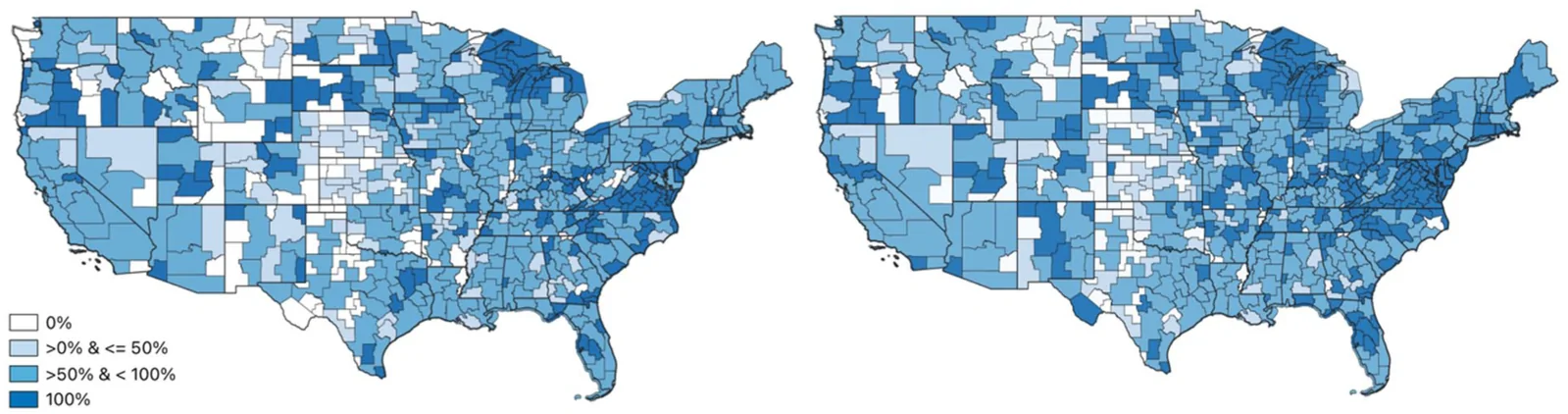
Distribution of health system penetration in 2016 (left) and 2023 (right). *Note*. The map was created using Quantum Geographic Information System (QGIS). For visual clarity, the geographic representation is limited to the contiguous United States.

**Table 1.** Descriptive Statistics of Sample, by Levels of Health System Penetration.

	Solo-hospital zones		Multi-hospital zones		
Characteristic	0%	100%	0%	>0%, <100%	100%
*N* (zone-year, %)	326 (47.7%)	358 (52.3%)	290 (8.4%)	2,185 (63.2%)	985 (28.5%)
% population aged 65+ (*SD*)	19.496 (6.275)	20.374 (4.979)	19.090 (4.843)	17.514 (3.586)	18.652 (3.655)
% adults aged 25+ high school graduate or higher	88.477 (6.461)	87.348 (5.868)	85.820 (7.453)	88.262 (4.684)	89.198 (4.214)
% population with private insurance	61.278 (11.902)	61.885 (9.773)	62.986 (10.217)	65.718 (8.312)	67.443 (7.482)
% population with Medicaid	22.177 (9.045)	23.080 (7.686)	20.279 (8.439)	21.054 (6.606)	20.495 (5.889)
Zone minority type					
High-Black	18 (5.5%)	24 (6.7%)	14 (4.8%)	230 (10.5%)	91 (9.2%)
High-Hispanic	35 (10.7%)	35 (9.8%)	71 (24.5%)	257 (11.8%)	46 (4.7%)
High-other	36 (11.0%)	28 (7.8%)	22 (7.6%)	39 (1.8%)	24 (2.4%)
Non-minority	237 (72.7%)	271 (75.7%)	183 (63.1%)	1,659 (75.9%)	824 (83.7%)
% population below poverty level	15.423 (7.322)	16.043 (5.828)	15.419 (5.372)	15.030 (4.556)	14.184 (4.342)
Number of hospitals in Zone	N/A	N/A	2.972 (1.191)	8.735 (10.708)	5.188 (4.493)
Herfindahl Hirschman Index (per 1,000 points)	N/A	N/A	4.596 (1.464)	3.914 (1.720)	5.828 (2.298)
Region					
Midwest	86 (26.4%)	74 (20.7%)	96 (33.1%)	754 (34.5%)	340 (34.5%)
Northeast	0 (0.0%)	24 (6.7%)	1 (0.3%)	159 (7.3%)	80 (8.1%)
South	86 (26.4%)	147 (41.1%)	106 (36.6%)	834 (38.2%)	435 (44.2%)
West	154 (47.2%)	113 (31.6%)	87 (30.0%)	438 (20.0%)	130 (13.2%)
Urban	19 (5.8%)	70 (19.6%)	15 (5.2%)	1,124 (51.4%)	574 (58.3%)
Year					
2016	55 (16.9%)	49 (13.7%)	65 (22.4%)	390 (17.8%)	129 (13.1%)
2018	63 (19.3%)	51 (14.2%)	60 (20.7%)	365 (16.7%)	151 (15.3%)
2020	51 (15.6%)	64 (17.9%)	43 (14.8%)	358 (16.4%)	174 (17.7%)
2021	53 (16.3%)	64 (17.9%)	37 (12.8%)	368 (16.8%)	170 (17.3%)
2022	52 (16.0%)	65 (18.2%)	40 (13.8%)	355 (16.2%)	180 (18.3%)
2023	52 (16.0%)	65 (18.2%)	45 (15.5%)	349 (16.0%)	181 (18.4%)

*Source*. Authors’ analysis of data.

*Note*. In solo-hospital zones, only region (*p* < .001) and urban-rural status (*p* < .001) differed significantly across categories. All other characteristics were not statistically significant. In multi-hospital zones, all characteristics differed significantly across categories (all *p* ≤ .001). *SD*: standard deviation. Kruskal–Wallis test for continuous variables. Pearson’s test for factor variables.

### Solo-Hospital Zones (Adjusted Analyses)

In solo-hospital zones, a larger older population and greater private insurance coverage were both associated with a higher probability that the only hospital in the zone was affiliated with a health system (Table 2). A 1-percentage-point (pp) increase in the proportion of the population living below the poverty level was associated with a 2.03-pp (95% confidence interval [CI] [0.23, 3.83]; *p* < .05) higher probability of system affiliation for the sole hospital in the zone.

**Table 2.** Logistic Regression Estimates.

Characteristics	Average marginal effect
% population aged 65+	2.08* (0.44, 3.72)
% adults aged 25+ high school graduate or higher	−2.24 (−4.51, 0.03)
% population with private insurance	2.69** (1.14, 4.24)
% population with Medicaid	1.27 (−0.20, 2.75)
Zone minority type	
Non-minority (Ref.)	
High-Black	−13.04 (−43.01, 16.93)
High-Hispanic	−11.28 (−41.38, 18.82)
High-other	24.95* (2.71, 47.19)
% population below poverty level	2.03* (0.23, 3.83)
Region	
Midwest (Ref.)	
Northeast	N/A
South	11.58 (−14.81, 37.97)
West	7.62 (−13.41, 28.65)
Urban	33.09*** (15.32, 50.86)
*N* (Zone-year)	684

*Source*. Authors’ analysis of data.

*Note*. Results are from a logistic regression model. Numbers are average marginal effects, expressed in percentage points, and were estimated using margins after model estimation. All Northeast observations had 100% system penetration, so Stata omitted the Northeast indicator because of perfect prediction. The Herfindahl–Hirschman Index and number of hospitals were excluded from the analysis. 95% confidence intervals are in parenthesis. Bold-faced values indicate statistical significance (*p* < .05).

* *p* < .05, **p < .01, ***p < .001.

### Multi-Hospital Zones (Adjusted Analyses)

Among zones with more than one hospital, a 1-pp increase in the proportion of adults aged 25 years or older with at least a high school diploma was associated with a 0.59-pp (95% CI [−1.15, −0.03]; *p* < .05) lower probability of having no health system penetration (Table 3). Consistent with the pattern, the beta regression component showed that a 1-pp increase in educational attainment was associated with a 0.68-pp (95% CI [0.12, 1.24]; *p* < .05) higher level of system penetration among zones with partial penetration. Relative to rural zones, urban zones had a 20.48-pp (95% CI [16.35, 24.61]; *p* < .001) higher level of system penetration. In the one-inflation component, both a higher proportion of older adults (1.77 pp; 95% CI [0.79, 2.75]; *p* < .001) and greater private insurance coverage (1.45 pp; 95% CI [0.47, 2.43]; *p* < .01) were associated with a greater probability of full penetration. A 1,000-point increase in the HHI was associated with a 7.75-pp (95% CI [6.36, 9.14]; *p* < .001) higher probability of full health system penetration.

**Table 3.** Zero-One Inflated Beta Regression Estimates.

	All zones			Rural zones			Urban zones		
Characteristics	Zero-inflate regression model (P(Y=0))	Beta regression model	One-inflate regression model (P(Y=1))	Zero-inflate regression model (P(Y=0))	Beta regression model	One-inflate regression model (P(Y=1))	Zero-inflate regression model (P(Y=0))	Beta regression model	One-inflate regression model (P(Y=1))
% population aged 65+	0.73** (0.18, 1.28)	−0.21 (−0.76, 0.35)	1.77*** (0.79, 2.75)	1.28* (0.20, 2.36)	−0.52 (−1.49, 0.46)	1.69** (0.47, 2.92)	−0.18 (−0.41, 0.05)	0.08 (−0.38, 0.54)	1.45 (−0.26, 3.16)
% adults aged 25+ high school graduate or higher	−0.59* (−**1.15**, −**0.03)**	0.68* (0.12, 1.24)	−0.82 (−2.10, 0.45)	−0.77 (−1.85, 0.31)	1.20** (0.33, 2.07)	−0.59 (−2.03, 0.85)	−0.23 (−0.55, 0.10)	0.42 (−0.39, 1.23)	−0.88 (−3.46, 1.70)
% population with private insurance	0.24 (−0.26, 0.73)	−0.32 (−0.82, 0.19)	1.45** (0.47, 2.43)	0.33 (−0.58, 1.23)	−0.53 (−1.27, 0.22)	2.30*** (1.15, 3.45)	0.28 (−0.11, 0.68)	−0.42 (−1.02, 0.18)	0.43 (−1.38, 2.23)
% population with Medicaid	−0.25 (−0.71, 0.21)	−0.13 (−0.51, 0.24)	0.83 (−0.04, 1.71)	−0.54 (−1.40, 0.32)	−0.24 (−0.78, 0.31)	1.35* (0.31, 2.38)	0.36* (0.04, 0.68)	−0.55** (−**0.89**, −**0.20)**	0.07 (−1.38, 1.53)
Minority type									
Non-minority (Ref.)									
High-Black	4.61 (−8.75, 17.97)	−2.68 (−7.49, 2.13)	3.14 (−8.08, 14.35)	5.27 (−18.93, 29.47)	−9.38 (−19.02, 0.25)	−4.81 (−20.79, 11.17)	2.69 (−5.67, 11.05)	2.46 (−0.93, 5.86)	6.75 (−7.91, 21.41)
High-Hispanic	10.30* (0.86, 19.74)	−0.39 (−5.68, 4.89)	2.79 (−12.16, 17.75)	20.57* (3.17, 37.96)	4.13 (−4.47, 12.73)	−8.76 (−26.15, 8.63)	−2.19** (−**3.50**, −**0.87)**	1.54 (−2.52, 5.61)	3.07 (−18.61, 24.75)
High-other	28.27** (8.88, 47.66)	−9.05 (−20.77, 2.66)	15.70 (−4.79, 36.19)	46.42** (15.98, 76.86)	−17.61 (−41.20, 5.99)	24.51* (0.13, 48.90)	−2.04** (-3.37, −**0.70)**	−1.31 (−8.02, 5.40)	3.67 (−19.91, 27.25)
% population below poverty level	−0.28 (−0.94, 0.37)	−0.11 (−0.83, 0.60)	−0.34 (−1.60, 0.93)	−0.48 (−1.68, 0.73)	0.12 (−1.00, 1.23)	1.04 (−0.60, 2.68)	0.52* (0.11, 0.94)	−0.09 (−0.89, 0.72)	−1.72 (−3.45, 0.01)
Number of hospitals in the zone	−6.69*** (−**9.02**, −**4.36)**	0.76** (0.24, 1.28)	−0.56 (−1.27, 0.14)	−11.02*** (−**15.05**, −**6.98)**	4.76*** (3.56, 5.95)	−0.90 (−3.37, 1.57)	−1.90** (−**3.30**, −**0.51)**	0.27 (−0.07, 0.61)	−1.17* (−2.14, -0.20)
Herfindahl Hirschman Index (per 1,000 points)	−3.42*** (-5.17, −1.67)	2.37*** (1.10, 3.64)	7.75*** (6.36, 9.14)	−5.97*** (−9.26, −2.69)	6.33*** (4.77, 7.88)	7.80*** (6.28, 9.32)	−0.96** (−1.67, −0.25)	0.32 (**−**0.84, 1.47)	5.87*** (3.50, 8.25)
Region									
Midwest (Ref.)									
Northeast	−5.66* (−11.01, −0.32)	7.10* (1.23, 12.98)	6.52 (**−**6.29, 19.32)	**−**8.75 (**−**19.06, 1.56)	7.75 (**−**1.14, 16.64)	**−**6.00 (**−**24.38, 12.39)	0.00 (0.00, 0.00)	1.90 (**−**2.18, 5.98)	14.60 (**−**3.03, 32.24)
South	1.49 (**−**4.24, 7.22)	5.49* (1.15, 9.83)	9.46 (**−**0.19, 19.11)	3.87 (**−**7.81, 15.55)	11.69** (3.70, 19.68)	8.19 (**−**5.29, 21.66)	1.13 (**−**0.06, 2.32)	**−**1.75 (**−**5.25, 1.76)	8.53 (**−**4.51, 21.57)
West	3.50 (**−**2.64, 9.64)	**−**1.71 (**−**6.37, 2.96)	**−**5.17 (**−**14.78, 4.45)	4.19 (**−**6.46, 14.85)	**−**1.05 (**−**8.24, 6.14)	1.26 (**−**9.48, 12.00)	14.41* (3.28, 25.53)	**−**1.27 (**−**5.10, 2.56)	**−**8.44 (**−**24.31, 7.42)
Urban	**−**5.27 (**−**11.36, 0.83)	20.48*** (16.35, 24.61)	16.42*** (9.31, 23.52)	N/A			N/A		
*N* (Zone-year)	3,460			1,747			1,713		

*Source*. Authors’ analysis of data.

*Note*. Results are from a zero-one inflated beta regression model. The zero-inflation component models the probability that *y* = 0, corresponding to no health system penetration. The proportion component uses beta regression with a logit link to model the conditional mean of the outcome for observations with 0 < *y* < 1, that is, zones with partial health system penetration. The one-inflation component models the probability that *y* = 1, corresponding to full health system penetration. Numbers are average marginal effects, expressed in percentage points, and were estimated using margins after model estimation. 95% confidence intervals are in parenthesis. Bold-faced values indicate statistical significance (*p* < .05).

* *p* < .05, **p < .01, ***p < .001.

In rural zones, relative to non-minority zones, high-Hispanic zones had a 20.57-pp (95% CI [3.17, 37.96]; *p* < .05) higher probability of having no health system penetration. Both a greater number of hospitals and a higher HHI were associated with a lower probability of having no system penetration. In contrast, these same characteristics were associated with higher levels of health system penetration in the beta regression component. In the one-inflation component, a higher proportion of older adults, greater private insurance coverage, and greater Medicaid coverage were each associated with a higher probability of full health system penetration.

In urban zones, relative to non-minority zones, high-Hispanic zones had a 2.19-pp (95% CI [−3.50, −0.87]; *p* < .01) lower probability of having no health system penetration. Relative to zones in the Midwest, zones in the West had a 14.41-pp (95% CI [3.28, 25.53]; *p* < .05) higher probability of having no health system penetration.

Results were generally similar, although some differences emerged when minority zones were defined relative to the state-specific rather than the national distribution (Supplemental Appendix II in the supplemental materials). In urban zones, relative to non-minority zones, high-Black zones had a 14.26-pp (95% CI [11.83, 16.68]; *p* < .001) higher probability of having no system penetration. In the beta regression component, high-Black zones had 4.53-pp (95% CI [1.86, 7.19]; *p* < .01) higher system penetration than non-minority zones. In the one-inflation component, high-Hispanic zones had a 17.11-pp (95% CI [3.42, 30.80]; *p* < .05) higher probability of full health system penetration than non-minority zones.

## Discussion

Integrated health systems have become a central feature of U.S. health care delivery and an increasingly important focus of policy and research. In this study, we examined the association between community characteristics and health system penetration, measured through ownership or joint management of general acute care hospital beds, across geographic zones. Given both the potential benefits and the potential harms that health systems may bring to the communities they serve, areas with high health system penetration as well as those with little or no penetration warrant attention.

We found a positive association of both hospital count and HHI with health system penetration, suggesting that health systems are more prevalent in markets that are both larger and more concentrated. A greater number of hospitals may create more opportunities for system expansion, particularly in rural areas. The positive association between HHI and health system penetration suggests that markets with greater system presence also tend to be more concentrated. Or if system-affiliated hospitals tend to have more beds, greater health system penetration may itself contribute to a higher measured HHI. This relationship is nevertheless consistent with recent evidence showing that one or two health systems controlled the entire market in nearly half of metropolitan areas in 2024 (Godwin et al., 2026). These raise concerns that greater health system penetration may reduce market competition, which could strengthen systems’ bargaining power and contribute to higher prices and costs for patients and payers (Cutler & Scott Morton, 2013). In addition, recent evidence suggests that greater market concentration may also have implications for equity, as its effects on quality of care appear to vary by patient race and ethnicity and payer (Adia et al., 2025). Taken together, these findings suggest that the consequences of greater health system penetration may extend beyond market structure alone, with potential implications for affordability, access, and equity across communities.

We also found that zones in the Northeast and South were more likely than those in the Midwest to have higher levels of system penetration. This differs somewhat from a prior study showing that physician consolidation within health systems was especially pronounced in metropolitan statistical areas in the Midwest and Northeast (Kimmey et al., 2021). One possible explanation is that the prior work focused on physician integration, whereas our study focuses on hospital ownership. Health systems may pursue different strategies when integrating hospitals than when integrating physician practices. Differences in the geographic units of analysis may also help explain the divergent findings. In addition, we found that higher educational attainment was associated with both a lower probability of no system penetration and a higher level of penetration. One possible explanation is that educational attainment is often associated with higher health literacy and greater engagement with health care (Coughlin et al., 2020; Stormacq et al., 2019). More educated populations may therefore be more likely to use preventive, specialty, and planned health care services (Rasu et al., 2015), creating stable demand and making these markets more attractive to health systems. At the same time, educational attainment may also proxy for broader community advantages, such as higher income levels and greater socioeconomic resources in the area, which could partly underlie this association.

Our stratified analyses further revealed that some factors were more strongly associated with health system penetration in urban areas, whereas others were more strongly associated in rural areas. In rural zones, greater insurance coverage and a higher proportion of older adults were associated with a higher probability of full system penetration. This finding is plausible given the financial vulnerability of rural hospitals (Carroll et al., 2024). Health systems may be more likely to enter rural markets with stronger insurance coverage because those markets offer greater financial viability. Moreover, a higher proportion of older adults may also imply a larger Medicare-covered population. This is consistent with the important role of Medicare for rural health care providers (McBride & Mueller, 2002), as Medicare payments are an important source of revenue for rural hospitals (Stensland & Domenella, 2025).

In urban zones, we found a complex pattern for high-Black communities when minority zones were defined using the state-specific distribution. Relative to non-minority zones, high-Black zones were more likely to have no system penetration at all. Prior research documenting persistent underinvestment in Black communities. For example, previous studies have shown that hospitals are more likely to discontinue services in areas with larger Black populations (Hsia & Shen, 2011; Hsia et al., 2012), and that communities with higher concentrations of Black residents tend to have fewer primary care physicians (Gaskin et al., 2012). However, we also found that among zones with some system presence, high-Black zones were associated with higher levels of penetration than non-minority zones. This suggests that patterns of investment across high-Black communities are not uniform. Some high-Black zones may be bypassed by health systems entirely, whereas others may experience larger system penetration relative to non-minority zones. This heterogeneity suggests that high-Black communities may be embedded in fundamentally different market environments, and future research is needed to better understand the factors that distinguish high-Black markets with health system presence from those without it. Those patterns were not observed when minority zones were defined using the national distribution. The state-specific definition identifies communities that are more racially concentrated relative to other communities within the same state and therefore may better capture within-state patterns of uneven investment.

Our study has important implications for both policy and future research. The lower probability of health system penetration in some high-Black zones raises concerns about underinvestment in those areas and potential disparities in health care access. Policymakers should consider targeted incentives and support mechanisms to encourage health systems to invest in underserved regions, promoting equitable access to high-quality care and supporting long-term health improvements in these communities. Given the distinct urban–rural dynamics we observed, regulatory approaches should be context-specific. For example, rural hospital mergers driven by financial distress may warrant regulatory flexibility, financial support, and service line maintenance requirements. Our finding that communities with higher health system penetration also tended to have lower levels of competition suggests the need for more stringent antitrust scrutiny aimed at preserving competition and limiting price growth. Finally, because the zero-inflation, beta, and one-inflation components did not all go in the direction that suggests the community characteristic has a significant association with system penetration, and because the findings for high-Black zones were particularly mixed, our results suggest that the relationship between community characteristics and health system penetration is complex. Future research is needed to better understand the distinct market conditions and mechanisms associated with no system presence, partial penetration, and full penetration. Additional work should also examine market dynamics more directly, including pre- and post-acquisition market environments and longitudinal changes in health system penetration.

Our study has several limitations. First, this study is descriptive and should not be interpreted as establishing causal relationships. In addition, because the analysis pooled community zone-years across the study period, the findings should be interpreted as broad associations between community characteristics and health system penetration during the study period rather than as changes associated with market entry or acquisition. Second, health system consolidation decisions are complex and influenced by many factors beyond geographic characteristics. For example, we do not account for system-level strategies or characteristics of health systems and affiliated hospitals, which are beyond the scope of this study but may be important determinants of market entry decisions. Third, although our community indicators provide meaningful proxies for community context, they may not fully capture the complexity of local market characteristics. Future research could incorporate additional measures, such as health insurance market competition, specialty service availability, or physician supply, to more precisely characterize the factors driving health system penetration. Fourth, our measure of system penetration focuses specifically on health system ownership or joint management of general acute care hospitals. However, system consolidation can also occur through other organizational arrangements. Future studies could explore these additional forms to provide a more complete picture of health system expansion.

## Conclusion

Health systems are not distributed evenly across markets, and the factors associated with greater penetration differ between rural and urban areas. Given the increasingly central role of health systems in care delivery, and the potential resources and benefits they may bring to communities, it is essential to ensure that health system consolidation does not exacerbate existing inequities in access to health care. Policymakers should closely monitor market competition and develop context-specific regulatory frameworks that protect underserved communities.

## Supplemental Material

sj-docx-1-mcr-10.1177_10775587261469029 – Supplemental material for Community Characteristics Associated With Health System Penetration Supplemental material, sj-docx-1-mcr-10.1177_10775587261469029 for Community Characteristics Associated With Health System Penetration by Haoyu Bi, Mark Louie Ramos, Jeannette A. Rogowski and Charleen Hsuan in Medical Care Research and Review
