# Supplementary material for: Community Characteristics Associated With Health System Penetration
Source: Med Care Res Rev. 2026 Sep 28;83(6):444–53. doi: 10.1177/10775587261469029 (PMC13622977; doi:10.1177/10775587261469029)
Supplement: Supplementary file 1 [file sj-docx-1-mcr-10.1177_10775587261469029.docx]

**Community Characteristics Associated with Health System Penetration**

**Supplemental Material**

**Appendix I**

Note. The analytic period spanned from 2016 to 2023, excluding 2017 and 2019, because Agency for Healthcare Research and Quality Compendium data were unavailable for those years.

CZ: community zone.

**Appendix II**

**Table S1. Logistic Regression Estimates.**

| Characteristics | Average marginal effect |
| --- | --- |
| % population aged 65+ | 1.14 (-0.50, 2.78) |
| % adults aged 25+ high school graduate or higher | -1.50 (-3.70, 0.70) |
| % population with private insurance | 1.40 (-0.23, 3.02) |
| % population with Medicaid | 0.59 (-0.95, 2.12) |
| Zone minority type |  |
| Non-minority (Ref.) |  |
| High-Black | -4.52 (-39.82, 30.79) |
| High-Hispanic | -15.14 (-49.16, 18.87) |
| High-other | -19.75 (-51.80, 12.30) |
| % population below poverty level | 1.76 (-0.20, 3.73) |
| Region |  |
| Midwest (Ref.) |  |
| Northeast | N/A |
| South | 3.27 (-21.93, 28.47) |
| West | 4.62 (-16.44, 25.68) |
| Urban | **33.79*** (15.60, 51.98)** |
| N (Zone-year) | 684 |

Source. Authors’ analysis of data.

Notes. Results are from logistic regression model. Numbers are average marginal effects, expressed in percentage points, and were estimated using margins after model estimation. All Northeast observations had 100% system penetration, so Stata omitted the Northeast indicator because of perfect prediction. The Herfindahl–Hirschman Index and number of hospitals were excluded from the analysis. 95% confidence intervals are in parenthesis.

*** p<.001, ** p<.01, * p<.05.

**Table S2. Zero-One Inflated Beta Regression Estimates.**

|  | All zones | | | Rural zones | | | Urban zones | | |
| --- | --- | --- | --- | --- | --- | --- | --- | --- | --- |
| Characteristics | Zero-inflate regression model (P(Y=0)) | Beta regression model | One-inflate regression model (P(Y=1)) | Zero-inflate regression model (P(Y=0)) | Beta regression model | One-inflate regression model (P(Y=1)) | Zero-inflate regression model (P(Y=0)) | Beta regression model | One-inflate regression model (P(Y=1)) |
| % population aged 65+ | 0.53 (-0.05, 1.11) | -0.15 (-0.70, 0.41) | **1.76*** (0.78, 2.73)** | 0.97 (-0.22, 2.16) | -0.63 (-1.59, 0.32) | **1.42* (0.17, 2.67)** | -0.23 (-0.55, 0.08) | 0.17 (-0.26, 0.60) | **1.72* (0.05, 3.40)** |
| % adults aged 25+ high school graduate or higher | **-0.80** (-1.36, -0.25)** | **0.73* (0.15, 1.31)** | -0.54 (-1.80, 0.73) | **-1.26* (-2.34, -0.19)** | **1.13* (0.25, 2.00)** | -0.50 (-1.93, 0.92) | **-0.31*** (-0.48, -0.15)** | 0.35 (-0.40, 1.10) | -0.57 (-2.93, 1.79) |
| % population with private insurance | -0.08 (-0.62, 0.45) | -0.31 (-0.83, 0.20) | **1.50** (0.50, 2.51)** | -0.24 (-1.20, 0.72) | -0.60 (-1.34, 0.15) | **2.15** (0.94, 3.37)** | 0.35 (-0.12, 0.82) | -0.45 (-1.05, 0.16) | 0.52 (-1.20, 2.24) |
| % population with Medicaid | -0.44 (-0.93, 0.04) | -0.15 (-0.53, 0.23) | 0.85 (-0.03, 1.73) | -0.87 (-1.78, 0.04) | -0.21 (-0.78, 0.36) | **1.34* (0.29, 2.39)** | 0.21 (-0.05, 0.47) | **-0.67*** (-1.02, -0.32)** | -0.09 (-1.50, 1.33) |
| Minority type |  |  |  |  |  |  |  |  |  |
| Non-minority (Ref.) |  |  |  |  |  |  |  |  |  |
| High-Black | -1.49 (-11.60, 8.61) | 2.30 (-1.42, 6.02) | -0.02 (-9.69, 9.64) | -8.79 (-25.65, 8.07) | -0.25 (-8.20, 7.71) | -12.05 (-27.12, 3.02) | **14.26*** (11.83, 16.68)** | **4.53** (1.86, 7.19)** | 7.57 (-4.28, 19.43) |
| High-Hispanic | -1.97 (-8.50, 4.56) | 1.93 (-2.68, 6.54) | 8.43 (-3.38, 20.25) | -3.32 (-15.53, 8.89) | 1.27 (-5.66, 8.21) | -10.47 (-25.68, 4.73) | **-2.13*** (-3.02, -1.25)** | 2.97 (-1.73, 7.67) | **17.11* (3.42, 30.80)** |
| High-other | 3.00 (-6.37, 12.38) | -3.69 (-9.95, 2.57) | -0.35 (-12.35, 11.66) | 5.48 (-11.69, 22.65) | **-12.66* (-23.02, -2.30)** | -11.06 (-25.55, 3.44) | **-2.08*** (-3.00, -1.17)** | 3.06 (-1.63, 7.76) | 5.48 (-10.80, 21.76) |
| % population below poverty level | -0.22 (-0.92, 0.49) | -0.17 (-0.90, 0.55) | -0.15 (-1.37, 1.08) | -0.36 (-1.65, 0.94) | -0.27 (-1.31, 0.77) | 0.92 (-0.62, 2.45) | **0.76*** (0.40, 1.12)** | -0.02 (-0.88, 0.84) | -1.40 (-3.07, 0.27) |
| Number of hospitals in the zone | **-7.05*** (-9.48, -4.62)** | **0.71** (0.19, 1.24)** | -0.56 (-1.26, 0.14) | **-12.06*** (-16.37, -7.76)** | **4.63*** (3.42, 5.84)** | -0.85 (-3.27, 1.57) | **-1.29*** (-1.96, -0.62)** | 0.26 (-0.07, 0.58) | **-1.26** (-2.19, -0.32)** |
| Herfindahl Hirschman Index (per 1,000 points) | **-3.73*** (-5.57, -1.88)** | **2.40*** (1.14, 3.67)** | **7.74*** (6.36, 9.13)** | **-6.68*** (-10.20, -3.17)** | **6.44*** (4.90, 7.98)** | **8.03*** (6.46, 9.60)** | **-0.61** (-1.06, -0.17)** | 0.40 (-0.74, 1.54) | **6.04*** (3.70, 8.39)** |
| Region |  |  |  |  |  |  |  |  |  |
| Midwest (Ref.) |  |  |  |  |  |  |  |  |  |
| Northeast | **-6.46* (-12.07, -0.85)** | **7.38* (1.54, 13.22)** | 6.38 (-5.98, 18.74) | -10.36 (-21.36, 0.65) | 7.59 (-1.31, 16.49) | -7.18 (-25.01, 10.66) | 0.98 (-3.27, 5.22) | 2.55 (-1.54, 6.65) | 15.41 (-2.02, 32.85) |
| South | -0.84 (-6.13, 4.45) | **5.21* (0.86, 9.57)** | **12.51* (2.74, 22.27)** | -0.82 (-11.02, 9.39) | **10.31* (2.18, 18.44)** | 7.79 (-5.75, 21.34) | **1.08* (0.09, 2.08)** | -0.70 (-3.81, 2.41) | **13.87* (1.48, 26.26)** |
| West | 4.44 (-2.40, 11.28) | -1.82 (-6.36, 2.71) | -3.56 (-12.74, 5.62) | 5.84 (-6.16, 17.85) | -1.08 (-8.17, 6.01) | 1.28 (-9.75, 12.31) | **28.08*** (19.77, 36.38)** | 0.11 (-3.37, 3.59) | -5.28 (-19.62, 9.06) |
| Urban | -4.77 (-10.75, 1.22) | **20.11*** (16.02, 24.19)** | **15.72*** (8.65, 22.80)** | N/A | | | N/A | | |
| N (Zone-year) | 3,460 | | | 1,747 | | | 1,713 | | |

Source. Authors’ analysis of data.

Notes. Results are from a zero-one inflated beta regression model. The zero-inflation component models the probability that y=0, corresponding to no health system penetration. The proportion component uses beta regression with a logit link to model the conditional mean of the outcome for observations with 0 < y < 1, that is, zones with partial health system penetration. The one-inflation component models the probability that y = 1, corresponding to full health system penetration. Numbers are average marginal effects, expressed in percentage points, and were estimated using margins after model estimation. 95% confidence intervals are in parenthesis.

*** p<.001, ** p<.01, * p<.05.
